# Familial Versus Non-Familial Vitiligo: Clinical Features, Anatomical Distribution, and Autoimmune Comorbidity from a Southern Taiwan Hospital

**DOI:** 10.3390/medicina61112040

**Published:** 2025-11-14

**Authors:** Ning-Sheng Lai, Hsiu-Hua Chang, Hui-Chin Lo, Ming-Chi Lu, Malcolm Koo

**Affiliations:** 1 Division of Allergy, Immunology and Rheumatology, Dalin Tzu Chi Hospital, Buddhist Tzu Chi Medical Foundation, Dalin, Chiayi 622401, Taiwan; 2 School of Medicine, Tzu Chi University, Hualien City, Hualien 970374, Taiwan; 3 Department of Medical Research, Dalin Tzu Chi Hospital, Buddhist Tzu Chi Medical Foundation, Dalin, Chiayi 622401, Taiwan; 4 Dalla Lana School of Public Health, University of Toronto, Toronto, ON M5T 3M7, Canada

**Keywords:** vitiligo, family history, autoimmune diseases, anatomic distribution, sex differences

## Abstract

*Background and Objectives*: Familial clustering and autoimmune multimorbidity are frequently observed in vitiligo. However, the clinical implications of a positive family history across generations remain unclear. In this study, a positive family history was defined as having at least one affected parent or grandparent. *Materials and Methods*: We retrospectively reviewed the electronic medical records of 972 adults with vitiligo who attended the rheumatology division in a regional teaching hospital in southern Taiwan between 2006 and 2022. Demographic characteristics, family history, clinical features, and autoimmune comorbidities were extracted from electronic medical records. Associations between family history and clinical parameters were assessed using logistic regression analyses adjusted for age and sex. *Results*: A total of 157 patients (16.2%) reported a family history, more often through parents than grandparents; maternal history was more common than paternal. Compared with those without a family history, affected families showed significantly younger age at diagnosis and a higher prevalence of lower-limb involvement. In adjusted models, family history was associated with greater odds of lower-limb involvement (adjusted odds ratio [aOR] 1.78, 95% confidence interval [CI] 1.22–2.58) and lower odds of eyebrow/eyelash depigmentation (aOR 0.39, 95% CI 0.16–0.92). Hashimoto thyroiditis was more frequent among familial cases (aOR 7.56, 95% CI 1.23–46.65). In sex-stratified analyses, associations were stronger in females, notably for lower-limb involvement (aOR 1.87), axillary depigmentation (aOR 2.33), and Hashimoto thyroiditis (aOR 11.27). *Conclusions*: Familial vitiligo shows earlier onset, distinct anatomical patterns, and increased thyroid autoimmunity, supporting systematic family-history assessment and targeted thyroid screening.

## 1. Introduction

Vitiligo is a common autoimmune pigmentary disorder characterized by the selective destruction of epidermal melanocytes, resulting in well-demarcated, chalk-white macules. Lesions often begin on the face and may extend to the neck, extremities, and trunk [1]. The condition occurs worldwide, with reported prevalence ranging from 0.06% to 2.3% across populations. The sex distribution is generally comparable, although some studies have noted a slightly higher frequency among females [2,3,4]. Familial clustering has been consistently observed, suggesting heritable predisposition, although most cases remain sporadic.

Building on this epidemiologic evidence, recent genetic studies have clarified that vitiligo results from a complex interplay between genetic susceptibility and environmental triggers. Heritability is high, and genome-wide association studies have identified multiple susceptibility loci that map predominantly to pathways involved in immune regulation, antigen presentation, and immune tolerance [5,6,7]. Variants within the major histocompatibility complex (MHC) class II region confer particularly strong risk and are associated with earlier disease onset [8]. Despite this strong genetic contribution, inheritance is non-Mendelian. Familial clustering is frequently reported, with approximately 20% to 30% of patients having an affected first-degree relative, corresponding to a seven- to tenfold increase in risk compared with the general population [9,10].

Beyond general heritability, investigations into lineage-specific transmission have produced inconsistent findings. Some studies suggest stronger paternal transmission, possibly influenced by imprinting effects, whereas others indicate maternal predominance [10,11]. Overall, these observations support a polygenic, multifactorial model rather than simple Mendelian inheritance, reflecting the complexity of genetic transmission across populations.

Sex-related immune and hormonal differences may further modulate these familial patterns. Estrogens enhance regulatory T- and B-cell responses, whereas androgens promote CD4+ and CD8+ T-cell activity [12]. Such immunologic contrasts may contribute to the observed sex variation in familial aggregation, clinical presentation, and autoimmune comorbidities.

Despite advances in understanding genetic and immunologic mechanisms, the phenotypic expression of vitiligo remains highly heterogeneous. Age at onset, anatomic distribution, hair depigmentation, and autoimmune comorbidities vary widely, and their relationships with positive family history remain insufficiently defined. Most prior studies have been conducted in dermatology settings or involved relatively small sample sizes, limiting their ability to compare clinical patterns and autoimmune profiles between familial and apparently sporadic cases. To address these uncertainties, we conducted a retrospective medical record review at a regional teaching hospital in southern Taiwan to compare demographic characteristics, anatomic distribution, hair depigmentation, and autoimmune comorbidities between patients with and without a family history of vitiligo. We hypothesized that familial vitiligo would be associated with earlier disease onset, distinctive anatomic distribution, and increased prevalence of autoimmune comorbidities.

## 2. Materials and Methods

### 2.1. Study Design and Setting

This retrospective study was conducted using electronic medical records from a regional teaching hospital in southern Taiwan. Patient encounters documented between 1 January 2006 and 30 November 2022 were screened for eligibility. At the time of our study enrollment, our hospital did not have a dermatologist on staff. Therefore, all patients with vitiligo were referred to the rheumatology division for evaluation and management. This arrangement enabled systematic assessment of potential autoimmune comorbidities by a multidisciplinary team with expertise in systemic autoimmune diseases, ensuring consistent documentation of relevant clinical and laboratory data. The study was approved by the Institutional Review Board of Dalin Tzu Chi Hospital, Buddhist Tzu Chi Medical Foundation (IRB No. B11104012-1).

### 2.2. Case Ascertainment

Patients with a recorded diagnosis of vitiligo were identified from electronic medical records. Vitiligo was diagnosed clinically by experienced rheumatologists based on characteristic depigmented macules and patches. Wood’s lamp examination was not routinely performed because the clinical features were typically sufficient for diagnosis. Data on age, sex, smoking status, disease duration, and family history were extracted for analysis.

No age restrictions were imposed, allowing for the inclusion of individuals across all age groups. Residence information was reviewed, and patients residing outside the southern or central regions of Taiwan were excluded from the study.

### 2.3. Ascertainment of Family History

In this study, family history was restricted to vertical relatives, defined as the presence of vitiligo in at least one parent or grandparent. We focused on these relatives because patients generally know the vitiligo status of parents and grandparents, whereas the status in siblings or offspring may be underreported due to age-dependent penetrance. Distinct generational strata also allow assessment of vertical transmission patterns across one and two generations without the added variability of horizontal relatives. Information on family history was obtained from patient self-report documented in medical records, and although this approach allows consistent data collection, it may be affected by recall accuracy.

### 2.4. Outcomes and Clinical Variables

Clinical severity at first visit was measured as a percentage of body surface area (BSA) affected, estimated using the Wallace Rule of Nines. In this method, the head and neck are estimated as 9%, each upper limb as 9%, each lower limb as 18%, the anterior trunk as 18%, and the posterior trunk as 18%, with the perineum representing 1% [13]. In addition, the patient’s own hand was used as the measuring device. The surface area of the palm, including the five digits, was considered approximately equivalent to 1% of the total body surface area. Data on autoimmune and cardiometabolic comorbidities, anatomic sites of vitiligo involvement, and the distribution of hair depigmentation were extracted from medical records, based on detailed documentation obtained during the initial clinical evaluation.

### 2.5. Statistical Analyses

All statistical analyses were performed using IBM SPSS Statistics, version 25.0.0.2 (Armonk, NY, USA). Continuous variables were summarized as mean and standard deviation (SD) or median with interquartile range (IQR), depending on distribution. Categorical variables were summarized as counts and percentages. Group comparisons by family-history status used the independent-samples *t* test or Mann–Whitney U test, and the χ^2^ test or Fisher’s exact test as appropriate. For multi-category variables, column proportions were compared using the z-test with Bonferroni correction. A two-tailed *p*-value < 0.05 was considered statistically significant.

To assess the degree of familial aggregation, we calculated the recurrence risk ratio (λ) for each relative class, defined as *λ_R_* = *K_R_*/*K*, where *K_R_* is the prevalence of vitiligo among relatives of probands in class *R*, and K denotes the population prevalence in Taiwan (0.064%) [4]. A higher λ indicates stronger familial aggregation; for example, λ = 4 implies that the prevalence among those relatives is approximately four times higher than in the general population. For each class, we calculated the proportion affected by dividing the number of affected relatives by the total number of relatives assessed, treating parents and grandparents as individual relatives (n = 972 per parent or grandparent class; n = 1944 for “any parent” and n = 3888 for “any grandparent”). We estimated 95% confidence intervals (CIs) for *λ_R_* by first computing exact (Clopper-Pearson) 95% CIs for *K_R_* and then dividing the limits by *K*. This approach provides conservative coverage when event counts are small, a condition in which normal (Wald) approximations can be unreliable.

Multiple logistic regression was used to assess whether associations observed in univariate analyses persisted after adjustment for sex and age. Overall models adjusted for sex and age; sex-stratified models adjusted for age only. We did not adjust for age at diagnosis or disease duration in models where these could lie on the causal pathway from family history to clinical features, to avoid collider bias. For example, patients with a family history may be diagnosed earlier, leading to longer disease duration by the index visit and a higher probability of certain findings. In addition, age at diagnosis can be influenced by access to care and symptom severity, so conditioning on it may open a spurious path between family history and clinical features through these unmeasured factors. Results were reported as adjusted odds ratios (aORs) with 95% CIs.

## 3. Results

### 3.1. Patient Characteristics

A total of 972 patients with vitiligo were included in this study, with 157 (16.2%) reporting a positive family history, defined as at least one affected parent or grandparent. The mean age was 48.5 years (SD 16.4). Women comprised 59.1% of the study participants. Sex distribution, body mass index category, and region of residence did not differ by family-history status. Mean age was significantly younger in those with a family history than those without (43.6 years vs. 49.5 years, *p* < 0.001). Current smoking was less common among those with a family history (12.1% vs. 18.9%, *p* = 0.042) (Table 1).

### 3.2. Clinical Characteristics

Table 2 presents the clinical characteristics of the patients. Age at diagnosis varied by family history. Compared with those without a family history, family-history positive patients were more often diagnosed before age 20 years (31.8% vs. 16.4%) and less often at 50–59 years (11.5% vs. 18.0%) or at 60 years or older (3.2% vs. 10.4%) (overall *p* < 0.001). Median BSA at first visit was 1.0% (IQR 2.6%) overall, with no difference by family history (1.5% vs. 1.0%, *p* = 0.186).

The face was the most frequent site (85.7%), followed by the upper limbs (73.7%), lower limbs (60.5%), trunk (45.9%), back (29.7%), and genital region (22.0%). Lower-limb involvement was more common among participants with a family history (70.1% vs. 58.7%, *p* = 0.007), while other site distributions were similar between groups.

Hair depigmentation was present in 42.0% of participants, without a between-group difference (45.2% vs. 41.3%, *p* = 0.368). By site, eyebrow or eyelash involvement was less frequent among those with a family history (3.8% vs. 8.6%, *p* = 0.042), whereas axillary involvement was more frequent (10.2% vs. 5.9%, *p* = 0.047). Scalp and genital hair depigmentation did not show differences between groups.

Comorbidities were broadly comparable across groups. Thyroid dysfunction occurred in 12.2% overall (*p* = 0.315). Hashimoto thyroiditis was uncommon but more frequent with a family history (1.9% vs. 0.2%, *p* = 0.008). Sjögren syndrome showed a trend toward lower prevalence in the family-history group that did not reach statistical significance (1.3% vs. 4.5%; *p* = 0.056). No cases of rheumatoid arthritis occurred among patients with a family history.

### 3.3. Distribution of Affected Relatives

Overall, 11.0% of probands reported an affected parent and 6.1% an affected grandparent (Table 3). Within the family-history positive subset, 68.2% reported an affected parent and 37.6% an affected grandparent. Maternal history was more frequent than paternal history (42.0% vs. 27.4%). Affected grandparents were reported across both paternal and maternal lineages, each contributing about 10 to 12% of family-history positive cases.

Table 4 presents the λ of vitiligo among parents and grandparents of probands, benchmarked to the Taiwanese population prevalence of 0.064% [4]. Among first-degree relatives, both showed marked excess prevalence relative to the population. The λ was 6.91 (95% CI 5.03–9.24) for fathers and 10.6 (95% CI 8.27–13.37) for mothers, with maternal estimates higher. When considering either parent affected, the λ was 2.73 (95% CI 1.60–4.35).

Grandparents also showed elevated aggregation, although of smaller magnitude than parents. Paternal grandparents had stronger estimates (grandfathers λ = 3.05, 95% CI 1.85–4.74; grandmothers λ = 4.74, 95% CI 3.62–6.09) than maternal grandparents (grandfathers λ = 1.45, 95% CI 0.66–2.73; grandmothers λ = 2.73, 95% CI 1.60–4.35). For any grandparent, λ = 2.37 (95% CI 1.81–3.05).

Overall, the pattern suggests a gradient by degree of relatedness, with the largest aggregation among mothers, intermediate aggregation among fathers, and more modest but still elevated aggregation among grandparents. However, estimates for grandparents should be interpreted with caution because of the small number of affected cases and potential differences in age and reporting accuracy.

### 3.4. Multiple Logistic Regression Analyses

**Figure 1:**
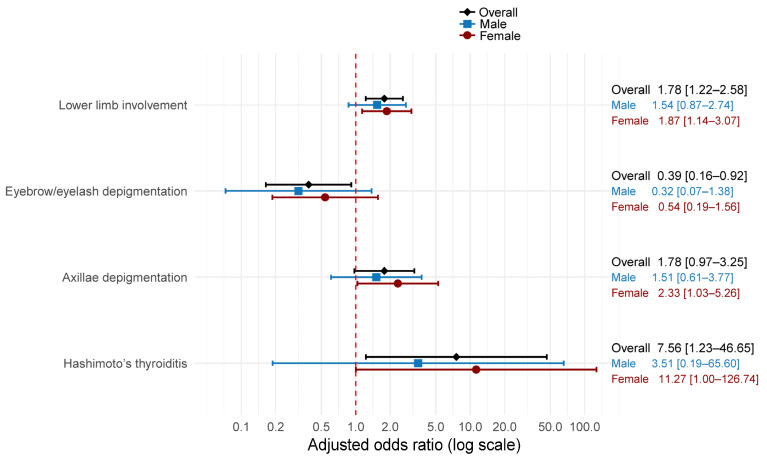
Forest plot of adjusted odds ratios and 95% confidence intervals for the association between family history of vitiligo and selected clinical characteristics, including lower limb involvement, eyebrow/eyelash depigmentation, axillae depigmentation, and Hashimoto’s thyroiditis. Overall models were adjusted for sex and age. Sex-stratified models were adjusted for age. The vertical dotted line at odds ratio = 1.0 represents the null value (no association).

Using a forest plot, Figure 1 presents adjusted odds ratios from logistic regression models for the association between family history and clinical characteristics. After adjustment for age and sex, family history remained significantly associated with greater odds of lower-limb involvement (aOR 1.78, 95% CI 1.22–2.58) and lower odds of eyebrow or eyelash depigmentation (aOR 0.39, 95% CI 0.16–0.92). Associations with axillary depigmentation were positive but not statistically significant (aOR 1.78, 95% CI 0.97–3.25). Hashimoto thyroiditis showed increased odds with wide uncertainty (aOR 7.56, 95% CI 1.23–46.65).

Sex stratification yielded similar directions of effect with reduced precision. Among males, point estimates were close to the null and nonsignificant for lower-limb involvement and axillary depigmentation. In contrast, the aOR moved further from the null for eyebrow or eyelash depigmentation (aOR 0.32, 95% CI 0.07–1.38). The association with Hashimoto thyroiditis was imprecise and not significant (aOR 3.51, 95% CI 0.19–65.60).

Among females, the association with lower-limb involvement (aOR 1.87, 95% CI 1.14–3.07) and axillary depigmentation (aOR 2.33, 95% CI 1.03–5.26) were significant associated with family history. Hashimoto thyroiditis also met significance thresholds with broad confidence intervals (aOR 11.27, 95% CI 1.00–126.74). Eyebrow or eyelash depigmentation was not statistically significant (aOR 0.54, 95% CI 0.19–1.56).

These adjusted analyses indicate that a reported family history of vitiligo is associated with greater lower-limb involvement and Hashimoto thyroiditis, but with lower odds of eyebrow or eyelash depigmentation. Associations appeared stronger in females, particularly for lower-limb involvement and Hashimoto thyroiditis, suggesting possible sex-specific differences.

## 4. Discussion

This hospital-based medical record study of 972 patients with vitiligo revealed the clinical characteristics and patterns of familial versus non-familial vitiligo. The finding that 16.2% of patients reported a positive family history, defined as the presence of vitiligo in at least one parent or grandparent, aligns with previous estimates indicating familial clustering occurs in approximately 20–30% of patients [9]. The results demonstrate several significant associations that warrant detailed discussion considering existing literature and clinical implications.

### 4.1. Basic and Clinical Characteristics

One intriguing finding was the lower prevalence of current smoking among patients with a positive family history. This contrasts with what might be expected if smoking were simply a shared environmental factor within families. Recent large-scale epidemiological data have suggested that smoking may be inversely associated with vitiligo risk. For instance, a Korean National Health Insurance Service study of over 23 million individuals reported that current smokers had a 31% lower risk of developing vitiligo compared with non-smokers (hazard ratio = 0.69, 95% CI 0.65–0.72), with a clear dose–response relationship. Proposed mechanisms include inhibition of monoamine oxidase, an enzyme upregulated in vitiligo that promotes oxidative stress [14].

These observations may help explain our findings, although the interpretation remains speculative. If smoking does reduce susceptibility to vitiligo, genetically predisposed individuals who smoke may have a lower probability of disease manifestation compared with their non-smoking counterparts. Consequently, smokers could be underrepresented among patients with both a family history and active disease. Nevertheless, this possibility should be regarded as a hypothesis rather than an inference, as causality cannot be determined from our data. It is also important to note that smoking has been associated with specific phenotypic patterns, such as a threefold higher risk of hand involvement [15], and its well-established adverse health consequences far outweigh any potential protective effects. Future studies should clarify whether this inverse relationship reflects biological modulation of disease pathways or differences in lifestyle and environmental exposures.

Familial cases tended to present at younger ages, consistent with previous studies and genetic susceptibility models [16,17]. This pattern supports the notion that genetic predisposition may accelerate disease onset, possibly through shared risk alleles with stronger effect sizes in multiplex families. An alternative explanation is that parents affected by vitiligo may recognize depigmentation earlier in their children, shortening the interval between onset and diagnosis. Both mechanisms, genetic susceptibility and ascertainment bias, are likely to operate concurrently, contributing to the younger age at diagnosis observed in familial cases.

### 4.2. Anatomical Distribution and Predilection Sites

The significantly higher prevalence of lower-limb involvement in patients with a family history represents an important clinical observation that remained significant after adjustment for age and sex. This association suggests that genetic factors may influence anatomical site susceptibility patterns. Lower limb vitiligo may be influenced by several mechanisms. From a mechanical perspective, the lower extremities are subject to repeated microtrauma from walking, tight clothing, and footwear, which could trigger Koebnerization in genetically susceptible individuals [18]. Moreover, the lower limbs have distinct vascular and neurological innervation patterns that may interact with genetic predisposition. Recent research has suggested that vitiligo distribution patterns, particularly in segmental forms, may correspond to underlying arterial territories, indicating that vascular factors could influence lesion localization [19].

The study also revealed contrasting patterns of hair depigmentation based on family history. Patients with familial vitiligo showed significantly lower rates of eyebrow or eyelash depigmentation, which remained significant after adjustment for age and sex. Conversely, axillary hair depigmentation was more frequent in the familial group, though this association did not maintain statistical significance after adjustment.

The differential involvement of hair-bearing areas suggests that genetic factors may influence the immune microenvironment of different follicular regions. Eyebrow and eyelash follicles exist within the periocular region, which has distinct immunological characteristics and may be subject to different regulatory mechanisms compared to axillary hair follicles. The periocular area has specialized immune privilege that may be differentially affected by genetic variants associated with familial vitiligo [20].

### 4.3. Autoimmune Comorbidities

One notable finding was the significant association between family history of vitiligo and Hashimoto thyroiditis, although the number of affected patients was small. After adjustment, the association remained significant but imprecise. This observation extends prior evidence, as numerous studies have established links between vitiligo and autoimmune thyroid disease, with Hashimoto thyroiditis being the most common thyroid disorder co-occurring with vitiligo [21,22].

Several biological mechanisms support this association. At the biochemical level, both melanocytes and thyrocytes utilize tyrosine as a precursor molecule, producing melanin and thyroxine, respectively [23]. Genetic studies further confirm shared susceptibility, identifying overlapping loci between vitiligo and autoimmune thyroid disease, including immune regulatory genes and organ-specific targets such as *TYR*, *TG*, and *TSHR*. Recent mechanistic reviews have also emphasized the role of antibody-mediated cellular activation and lipid raft–dependent signaling in autoimmune pathogenesis, which may represent additional shared pathways linking vitiligo with thyroid autoimmunity [24]. An autoimmunity susceptibility locus (AIS1) on chromosome 1 has also been described in families with both vitiligo and Hashimoto thyroiditis [21].

The stronger association observed in patients with a family history of vitiligo suggests that common genetic or familial susceptibility factors may predispose to both conditions within the same families. This interpretation is consistent with the broader concept of autoimmune clustering, in which shared pathways increase the likelihood of multiple autoimmune manifestations among genetically predisposed individuals. Clinically, these findings support the importance of screening and surveillance for thyroid autoimmunity in patients with familial vitiligo [25].

### 4.4. Inheritance Patterns and Sex Differences

In our study, maternal history was more frequent than paternal history (42.0% vs. 27.4% of family-history–positive patients), corresponding to higher familial aggregation for mothers (λ = 10.6) than for fathers (λ = 6.91). However, this difference did not reach statistical significance, as the confidence intervals overlapped. This pattern differs from several prior reports that found stronger paternal transmission, such as a Chinese study of 1136 patients that reported a higher λ for fathers (3.88) than mothers (1.79) [10], and an Indian study of 245 families that observed paternal transmission in 57.5% of cases compared with 42.5% for maternal transmission [11]. Such discrepancies across populations may reflect differences in genetic background, epigenetic imprinting, environmental exposures, or reporting biases, suggesting that parent-of-origin effects in vitiligo remain uncertain.

The multiple regression analysis revealed important sex-specific differences in clinical associations. Among females, family history was significantly associated with lower-limb involvement (aOR = 1.87), axillary depigmentation (aOR = 2.33), and Hashimoto thyroiditis (aOR = 11.27). In contrast, these associations were attenuated and nonsignificant in males.

Figure 1 illustrates these sex differences, with females showing consistently stronger associations across multiple clinical features compared to males. This pattern suggests that genetic counseling and risk assessment may need to account for patient sex when discussing familial vitiligo patterns.

This sex-specific pattern may reflect hormonal influences on disease expression and immune regulation. Estrogens can protect melanocytes from oxidative stress [26] and influence both innate and adaptive immune pathways [27]. The stronger associations observed among females with a family history suggest that hormonal factors modulate the penetrance or expressivity of genetic susceptibility variants in vitiligo.

Finally, while λ values also suggested aggregation among grandparents, these estimates were based on a small number of affected individuals and should therefore be interpreted with caution due to limited precision and potential reporting bias.

Taken together, these findings have direct implications for clinical management. Recognizing familial clustering and sex-specific differences may help clinicians identify patients at higher risk for autoimmune comorbidities, particularly thyroid disease, and guide counseling regarding prognosis and inheritance. These practical considerations are further elaborated in the following section.

### 4.5. Clinical Implications

Based on these findings, several practical recommendations emerge for clinical practice. First, clinicians should take a structured family history in all patients with vitiligo, specifically documenting parental and grandparental status. This information can inform prognosis, treatment selection, and family counseling. Second, patients with a positive family history, particularly females, should undergo baseline thyroid function assessment given the elevated risk of Hashimoto thyroiditis. Third, counseling families about vitiligo inheritance should emphasize that while familial aggregation exists, absolute risks remain modest for most relatives. Fourth, treatment approaches may need to consider anatomical predilection patterns in familial cases. The increased propensity for lower-limb involvement in familial vitiligo may influence treatment selection, as extremity lesions often respond less favorably to topical therapies and may benefit from early initiation of phototherapy or combination regimens [28].

Beyond vitiligo, familial aggregation has also been investigated in other chronic inflammatory skin diseases. In hidradenitis suppurativa, familial cases demonstrate a distinct clinical phenotype compared with sporadic disease, consistent with emerging genotype-phenotype links and substantial genetic heterogeneity [29]. In psoriasis, registry data indicate that a positive family history is associated with earlier onset and a higher prevalence of psoriatic arthritis, whereas overall severity measures may be similar [30]. In alopecia areata, a recent systematic review of 67 studies revealed that 17.6% of affected individuals have a positive family history of the condition and a significantly higher prevalence among first-degree relatives. Moreover, 9.6% of families of individuals with alopecia areata exhibited comorbid autoimmune or related conditions [31]. Together, these patterns support the concept that familial aggregation in autoimmune skin disease can influence age at onset, comorbidity profile, and selected clinical features, which aligns with our findings in vitiligo.

### 4.6. Limitations

This study has several limitations. First, as a retrospective, single-center study conducted in a regional hospital in southern Taiwan, the generalizability of our findings may be limited. Region-specific factors, such as environmental exposures, genetic background, or healthcare-seeking patterns, could influence the observed clinical and familial characteristics. Second, family history was obtained from patient self-report documented in medical records and limited to parents and grandparents. Nevertheless, our approach is consistent with prior studies that emphasized first- and second-degree vertical relatives as the most clinically informative categories of family history. Third, the assessment of disease severity in this study was limited to body surface area involvement at the first visit. Standardized indices of activity or progression were not available in retrospective records. Future prospective studies incorporating validated measures, such as the Vitiligo Area Scoring Index (VASI) [32], would allow more detailed evaluation of disease activity and progression in relation to family history. Fourth, the recurrence risk ratio (λ) for grandparents used a population prevalence denominator that does not account for age structure, so estimates should be interpreted cautiously. Finally, the cross-sectional design prevents assessing temporal relationships between family history and clinical features, limiting inferences about causality and disease progression patterns.

## 5. Conclusions

This analysis of familial versus non-familial vitiligo reveals several important clinical patterns that have implications for patient care and genetic counseling. Patients with a positive family history demonstrate earlier age at diagnosis, increased lower-limb involvement, distinctive hair depigmentation patterns, and elevated risk of Hashimoto thyroiditis, with particularly strong associations observed in females. The protective association with smoking requires further investigation, but should not influence clinical recommendations given smoking’s overall health risks.

These findings support the polygenic, multifactorial inheritance model of vitiligo while showing specific clinical features that may help identify patients at higher risk for certain disease patterns or comorbidities. The sex-specific differences in clinical associations suggest that hormonal factors may modify genetic susceptibility, warranting consideration in both research designs and clinical practice.

For clinical practice, structured family-history assessment, targeted thyroid screening in familial cases, and recognition of lower-limb predilection patterns may facilitate more personalized management of vitiligo. Continued research integrating genetic, immunologic, and clinical perspectives is needed to further clarify familial and sex-specific mechanisms underlying disease variability.

## Figures and Tables

**Table 1:** Basic characteristics of patients with familial and non-familial vitiligo (N = 972).

Variable	Total, n (%)	Family History of Vitiligo, n (%)		*p*
	972 (100)	Yes 157 (16.2)	No 815 (83.8)	
Age (years) mean (standard deviation)	48.5 (16.4)	43.6 (16.1)	49.5 (16.3)	<0.001
Sex				0.510
Male	398 (40.9)	68 (43.3)	330 (40.5)	
Female	574 (59.1)	89 (56.7)	485 (59.5)	
Body mass index category (kg/m^2^)				0.455
Normal (18.5 ≤ BMI < 24)	485 (49.9)	83 (52.9)	402 (49.3)	
Underweight (<18.5)	62 (6.4)	12 (7.6)	50 (6.1)	
Overweight (24 ≤ BMI < 27)	255 (26.2)	41 (26.1)	214 (26.3)	
Obese (≥27)	170 (17.5)	21 (13.4)	149 (18.3)	
Region of residence				0.299
Southern Taiwan	508 (52.3)	88 (56.1)	420 (51.5)	
Central Taiwan	464 (47.7)	69 (43.9)	395 (48.5)	
Smoking status				0.042
Yes	173 (17.8)	19 (12.1)	154 (18.9)	
No	799 (82.2)	138 (87.9)	661 (81.1)	

**Table 2:** Clinical characteristics of patients with familial and non-familial vitiligo (N = 972).

Variable	Total, n (%)	Family History of Vitiligo, n (%)		*p*
	972 (100)	Yes 157 (16.2)	No 815 (83.8)	
Age at vitiligo diagnosis (years)				<0.001
<20	184 (18.9)	50 (31.8) ^a^	134 (16.4) ^b^	
20–29	140 (14.4)	25 (15.9) ^a^	115 (14.1) ^a^	
30–39	193 (19.9)	26 (16.6) ^a^	167 (20.5) ^a^	
40–49	200 (20.6)	33 (21.0) ^a^	167 (20.5) ^a^	
50–59	165 (17.0)	18 (11.5) ^a^	147 (18.0) ^b^	
≥60	90 (9.3)	5 (3.2) ^a^	85 (10.4) ^b^	
BSA at first visit (%), median (IQR)	1.0 (2.6)	1.5 (3.1)	1.0 (2.6)	0.186
Predilection sites				
Face	833 (85.7)	137 (87.3)	696 (85.4)	0.542
Trunk	446 (45.9)	80 (51.0)	366 (44.9)	0.164
Upper limb	716 (73.7)	120 (76.4)	596 (73.1)	0.389
Lower limb	588 (60.5)	110 (70.1)	478 (58.7)	0.007
Back	289 (29.7)	54 (34.4)	235 (28.8)	0.163
Genital area	214 (22.0)	33 (21.0)	181 (22.2)	0.742
Hair depigmentation	408 (42.0)	71 (45.2)	337 (41.3)	0.368
Hair depigmentation sites				
Scalp	131 (13.5)	21 (13.4)	110 (13.5)	0.968
Eyebrows/eyelashes	76 (7.8)	6 (3.8)	70 (8.6)	0.042
Axillae	64 (6.6)	16 (10.2)	48 (5.9)	0.047
Genital area	169 (17.4)	32 (20.4)	137 (16.8)	0.279
Comorbidities				
Thyroid dysfunction	119 (12.2)	23 (14.6)	96 (11.8)	0.315
Thyroid nodules	39 (4.0)	8 (5.1)	31 (3.8)	0.450
Hashimoto thyroiditis	5 (0.5)	3 (1.9)	2 (0.2)	0.008
Rheumatoid arthritis	6 (0.6)	0 (0.0)	6 (0.7)	0.281
Sjögren’s syndrome	39 (4.0)	2 (1.3)	37 (4.5)	0.056
Systemic lupus erythematosus	11 (1.1)	1 (0.6)	10 (1.2)	0.522
Diabetes mellitus	48 (4.9)	6 (3.8)	42 (5.2)	0.481
Heart disease	31 (3.2)	5 (3.2)	26 (3.2)	0.997
Hypertension	128 (13.2)	16 (10.2)	112 (13.7)	0.228
Hyperlipidemia	56 (5.8)	9 (5.7)	47 (5.8)	0.986
Cancer history	30 (3.1)	2 (1.3)	28 (3.4)	0.208

BSA = body surface area; values summarized as median and interquartile range (IQR). Thyroid dysfunction refers to non-autoimmune hyper- or hypothyroidism. *p* values from *t*-test or Mann–Whitney U for continuous variables and χ^2^ or Fisher’s exact for categorical variables. Superscript letters denote column comparisons (*z*-test for column proportions with Bonferroni corrections), with different superscripts indicating significant differences at *p* < 0.05.

**Table 3:** Distribution of family history of vitiligo among parents and grandparents of probands (N = 972).

Relationship to Proband with Vitiligo	Vitiligo		
	n	% of Total	% of Family-History Positive
Any parent or any grandparent	157	16.2	100.0
Any parent	107	11.0	68.2
Father	43	4.4	27.4
Mother	66	6.8	42.0
Any grandparent	59	6.1	37.6
Paternal grandfather	17	1.7	10.8
Paternal grandmother	19	2.0	12.1
Maternal grandfather	9	0.9	5.7
Maternal grandmother	17	1.7	10.8

**Table 4:** Recurrence risk ratio (λ) of vitiligo among parents and grandparents of probands.

Relationship to Proband	Total Number	Vitiligo	Recurrence Risk Ratio, λ (95% CI)
Parents			
Father	972	43	6.91 (5.03–9.24)
Mother	972	66	10.6 (8.27–13.37)
Either parent	1944	107	2.73 (1.60–4.35)
Grandparents—paternal			
Grandfather	972	17	3.05 (1.85–4.74)
Grandmother	972	19	4.74 (3.62–6.09)
Either paternal grandparent	1944	35	2.81 (1.96–3.90)
Grandparents—maternal			
Grandfather	972	9	1.45 (0.66–2.73)
Grandmother	972	17	2.73 (1.60–4.35)
Either maternal grandparent	1944	25	2.01 (1.30–2.96)
Any grandparent	3888	59	2.37 (1.81–3.05)

## Data Availability

The data presented in this study are available on request from the corresponding author.

## Abbreviations

The following abbreviations are used in this manuscript:

aOR	Adjusted odds ratio
CI	Confidence interval
IQR	Interquartile range
λ	Recurrence risk ratio
SD	Standard deviation
